# Emerging frontiers in protein structure prediction following the AlphaFold revolution

**DOI:** 10.1098/rsif.2024.0886

**Published:** 2025-04-16

**Authors:** Martin Luke Rennie, Michael R. Oliver

**Affiliations:** ^1^ School of Molecular Biosciences, University of Glasgow, Glasgow, UK; ^2^ MRC-University of Glasgow Centre for Virus Research, Glasgow, UK

**Keywords:** AlphaFold, protein structure prediction, protein–protein interactions, co-evolution, conformational changes, biomolecular interactions

## Abstract

Models of protein structures enable molecular understanding of biological processes. Current protein structure prediction tools lie at the interface of biology, chemistry and computer science. Millions of protein structure models have been generated in a very short space of time through a revolution in protein structure prediction driven by deep learning, led by AlphaFold. This has provided a wealth of new structural information. Interpreting these predictions is critical to determining where and when this information is useful. But proteins are not static nor do they act alone, and structures of proteins interacting with other proteins and other biomolecules are critical to a complete understanding of their biological function at the molecular level. This review focuses on the application of state-of-the-art protein structure prediction to these advanced applications. We also suggest a set of guidelines for reporting AlphaFold predictions.

## 1. Protein structure prediction and the build-up to AlphaFold

### 1.1. Fundamental role of protein structures

**Figure 1.**
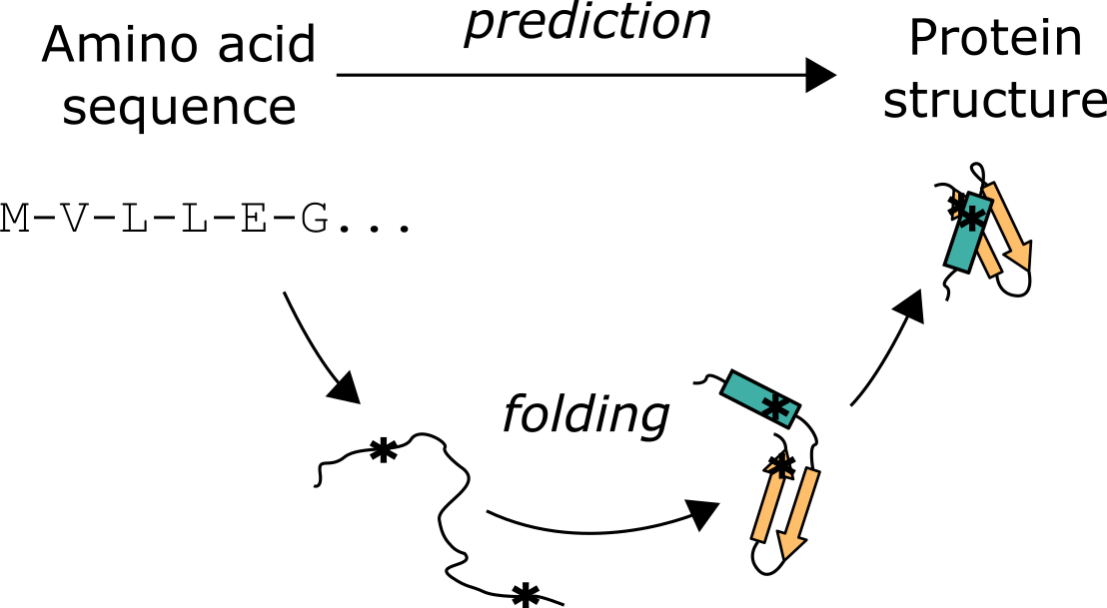
Protein folding versus structure prediction. Residues distal in the amino acid sequence (*) can end up proximal in the folded structure.

Proteins are polymers of amino acids and are involved in all processes of life, from enzymes that catalyse metabolic reactions, to motors that convert chemical potential energy into mechanical force, to cytoskeletal highways that facilitate transport. The diversity of these functions is achieved by their varied amino acid sequences. Twenty chemically distinct amino acids can be incorporated into linear polymers of hundreds, even thousands of units long. The first experimental protein structures determined in the 1950s and 1960s showed these linear polymers adopt complex folded chains [1]. Strikingly, amino acid residues distal in the sequence can be spatially proximal in the folded structure (figure 1). Despite this complexity, the molecular view of biology that protein structures enable creates a crucial interface at several disciplines. Chemically, structures can provide a basis for enzymatic reactions and help inform rational drug development. Clinically, structures can rationalize patient mutations in amino acid sequences. Experimentally, protein structures can integrate with other methods like mass spectrometry and biochemical assays to provide a more complete understanding of protein function.

Advances in experimental techniques have led to thousands of atomic models of proteins with distinct folds, highlighting the complexity of this class of biomolecules. Large-scale structural genomics projects have contributed significantly to this expansion of available structures [2]. However, experimental methods remain time-consuming, generally requiring isolation of the protein(s) of interest and using expensive X-ray sources, electron sources or high-field magnets, with extensive optimization required throughout the process. Technological advances have massively reduced the cost to sequence genomes, leading to identification of millions of different protein sequences. This has created a large disparity between the numbers of sequences versus structures that are available, and reliable methods to compute atomic models from amino acid sequences have been a goal for over 50 years.

Proteins attain their three-dimensional structure through a folding process, but this is far from straightforward. Even small protein polymers have huge degrees of freedom in their atomic geometry giving rise to vast spaces of potential conformations, yet proteins fold on biological timescales by exploring only a small subset of possible conformations (Levinthal’s paradox) [3]. Furthermore, the folding process is impacted by cellular factors such as (i) chaperones, other proteins that regulate folding [4], or (ii) pauses in translation driven by the RNA sequence encoding the protein [5,6], or even (iii) interaction with the ribosome itself [6]. As such the problem of deriving a general protein folding mechanism is not necessarily reducible to the amino acid sequence alone.

Protein structure prediction—the computation of protein structures from amino acid sequences—represents a distinct challenge from a complete understanding of the folding process itself [7]. This does not necessarily require consideration of the folding process and so while a solution to the protein folding problem encompasses a solution to protein structure prediction, the reverse is not necessarily true. The structure prediction field has greatly benefitted from the Critical Assessment of Methods of Protein Structure Prediction (CASP), a biannual competition that rigorously tests different structure prediction approaches [8]. Early CASP competitions recognized two prediction scenarios that reflect the fact that many proteins share similar folds. When experimental structures exist for proteins with similar folds (homologues), these can serve as templates to guide prediction—an approach termed template-based modelling. The more challenging scenario occurs when no such structural homologues are available, requiring ‘free modelling’ of the structure from sequence alone. CASP’s doubly blinded format, using carefully selected recent experimental structures as targets, provides an excellent framework for validating both approaches. Various methods have been developed to tackle these and other structure prediction tasks; however, we focus on a subset of the free modelling approaches leading to the state-of-the-art prediction tools that have revolutionized structural biology.

### 1.2. The evolution of protein structure prediction

**Figure 2.**
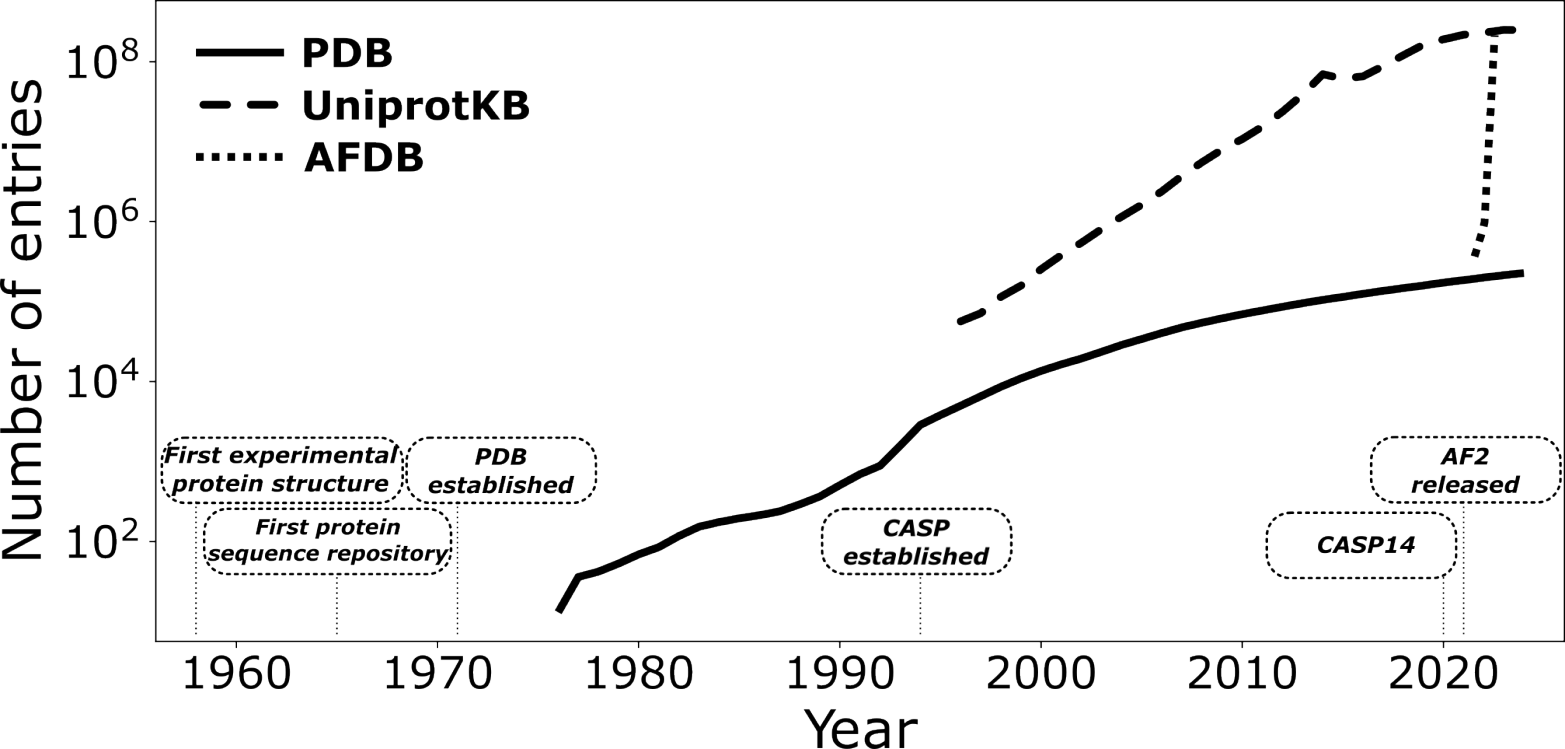
Database sizes over time and key developments in protein structure prediction. PDB entries as at 20 November 2024 (https://www.rcsb.org/stats/growth/growth-released-structures). UniProtKB entries as at 23 November 2024 (https://ftp.uniprot.org/pub/databases/uniprot/previous_releases/). AFDB entries from Varadi *et al*. [9].

The goal to predict protein structures from sequences originated shortly after the first experimental structures were determined; however, it took many decades and a convergence of numerous tools and databases to yield methods for general and accurate protein structure prediction (figure 2). The cornerstone of modern protein structure prediction is the concept of amino acid co-evolution [10–12]. In the 1980s, it was appreciated that there is selection pressure to maintain mutually compatible interactions within the protein structure, i.e. protein structure and function constrain the tolerance of the amino acid sequence to mutations [10]. However, it was not until the 1990s that the converse was formally proposed—analysis of mutations that occur naturally can be used to infer protein structure [11,12]. The correlations between amino acid positions, known as evolutionary couplings, can be determined by comparison against homologous sequences using multiple sequence alignments (MSAs). These couplings reflect co-evolution of amino acid positions and can be used to infer direct residue–residue contact probabilities, even between residues that are distal in the amino acid sequence.

Multiple methods have been used to extract coupling information from MSAs. Relatively simple correlation metrics were initially used with limited success [13]. This is because indirectly contacting residues can also co-evolve leading to false-positive predicted interactions [14]. To account for this, more advanced statistical analyses were used that distinguish between direct and indirect coupling [14–19]. The potential of this approach was first observed in CASP11 where a target more than 250 amino acid residues long, with many homologous sequences but no homologous structures available, was predicted with good precision [20].

The final technical advancement came from the application of neural networks to evolutionary coupling and structure prediction [21–25]. Several key developments in this field, particularly regarding deep learning, pushed protein structure prediction into broad practical utility. In 2012, the importance of large networks with many layers for pattern recognition was demonstrated for image classification [26]. In 2017, the transformer deep-learning architecture was introduced, exploiting an attention mechanism to capture long-range dependencies in sequences and facilitating massive parallel processing of sequence data [27]. However deep learning still requires a wealth of data to learn patterns, and extensive MSAs are required for each protein that is to be predicted.

Alongside these method developments, databases of protein sequences and structures have expanded by orders of magnitude (figure 2). The first repository of protein sequences was released in the 1960s [28] and since then a number of databases have been established [29–31]. Algorithms to rapidly search these vast sequence databases and assemble MSAs were also developed [32–34]. These developments allow generation of extensive MSAs for many proteins from many species. The Protein Data Bank (PDB) was established in 1971 as a repository of experimentally determined protein structures [35]. This database has grown to over 200 000 structures, although not all are unique proteins. The PDB also holds experimental and model statistics, which allows identification of high-quality models. This database provides an excellent source of information on the geometry amino acids adopt within protein structures. Both sequence and structure databases are critical for the application of deep learning to protein structure prediction.

The culmination of all these advancements occurred in CASP14 when AlphaFold2 (AF2) from the DeepMind team predicted structures of most of the novel targets with accuracies approaching experimental uncertainty [36,37]. AF2 made several key advances, including using an end-to-end deep neural network that simultaneously processes co-evolutionary information through a specialized transformer (Evoformer) and amino acid geometry through a structural module. AF2 also incorporated the use of homologous structures from the PDB as templates to initialize residue–residue contacts; however, these templates may have a minor effect on the quality of the predictions, particularly for sequences with deep MSAs and in many instances templates can be ignored.

It is worth noting that RoseTTAFold from the Baker group was subsequently developed and produces predictions approaching the accuracy of AF2 [38]. Protein language models have also been utilized to predict protein structures, the most powerful of which has been ESMFold [39,40]. These systems do not require MSAs and instead use a single sequence. While AF2 predictions that incorporate MSAs yield better predictions in general [39], for sequences with fewer identifiable homologues that generate shallow MSAs, ESMFold can outperform AF2 [41]. Furthermore, it has been suggested that protein language models have memorized motifs derived from co-evolutionary information [42], underscoring the importance of this information.

### 1.3. Accessibility of AlphaFold

Together with the release of AF2 code (https://github.com/google-deepmind/alphafold), predictions of proteomes of several well-studied organisms have been made publicly available through the European Bioinformatics Institute and DeepMind teams in the AlphaFold Database (AFDB) [43]. This has since been expanded to over 200 million predictions [9]. Although virus structures were not included in the AFDB, several groups have systematically applied protein structure prediction to viral proteomes (table 1) [41,43–45].

**Table 1.** Databases of predicted protein structures generated through large-scale computational prediction.

database	size	species	prediction type	website
AFDB [9]	>214 000 000	various (excluding viruses)	monomers	https://alphafold.ebi.ac.uk/
Big Fantastic Virus Databases [44]	>351 000	various viruses	monomers	https://bfvd.foldseek.com/
Nomburg *et al*. [45]	>67 000	various viruses	monomers	https://www.modelarchive.org/doi/10.5452/ma-jd-viral
Viro3D [43]	>85 000	various viruses	monomers	https://viro3d.cvr.gla.ac.uk/
Predictomes [46]	>47 000	*Homo sapiens*	pairwise PPIs	https://predictomes.org/
Computed human protein–protein interactome [47]	>18 000	*Homo sapiens*	pairwise PPIs	http://prodata.swmed.edu/humanPPI
FlyPredictome [48]	>106 000	*Drosophila*	pairwise PPIs	https://www.flyrnai.org/tools/fly_predictome/web/

Advancing the utility of these huge structural databases, Foldseek has been developed for rapid structural searches [49] and Foldmason for multiple structural alignments [50]. A major advance in accessibility of AF2 for bespoke purposes was aided by the MMSeq2 sequence search tool, to facilitate rapid MSA generation [34], and the Google Colab computing environment providing access to GPU compute necessary to run AF2. These are nicely tied together through ColabFold [51].

### 1.4. Impact of AlphaFold

A striking demonstration of the usefulness of structural predictions from state-of-the-art tools is their application to experimental structures. The most definitive example is that subtle errors in experimental structures, where the amino acid sequence in the model is offset from what it should be, known as sequence register errors, can be identified through discrepancies between experimental models and prediction models [49]. In these cases, the prediction frequently provides better local agreement with the experimental data than the human-built model [50]. Predictions can be used as base models to accelerate modelling of experimental structural data [51] and have also been used to solve the phase problem in protein crystallography [52]. The large-scale prediction efforts for viruses have led to the identification of novel protein folds [44,45], construction of structure-based virus phylogenies and elucidation of virus-host evolution [41].

The initial target of AlphaFold was individual protein monomers; however, proteins do not function in isolation; they interact with other proteins, nucleic acids and small molecules. The success of AlphaFold in predicting protein monomers has driven a shift towards structure prediction of these interactions. Protein–protein interactions (PPIs) also constrain mutations in protein sequences, i.e. evolutionary couplings can arise between amino acid positions from multiple different proteins. It is not surprising that there was almost immediate interest in applying AF2 to PPIs and within a few months of the initial release of AF2, AF2-multimer was released allowing multiple sequences to be incorporated into a single prediction [53]. For interactions involving two proteins, this is achieved through paired MSAs involving orthologues of protein pairs that facilitate extraction of evolutionary couplings between the proteins [53–55]. Several databases for pairs of interacting proteins have been developed, enhancing the accessibility of PPI predictions (table 1) [46–48]. In addition to modelling PPIs, protein interactions with nucleic acids, small molecules and post-translational modifications are also important targets for structural biology. AlphaFold3 (AF3), Chai-1, Boltz-1 and RosettaFold-All-Atom have been developed to extend predictions to include these molecules and modifications [56–59]. The code for all these models has been made available, and AF3 and Chai-1 have dedicated webservers for running predictions. At the time of writing, the AF3 server allows users 20 predictions per day, each prediction up to 5000 tokens (one token per polymer unit and one token per atom in non-polymers), while the Chai-1 server allows 25 predictions per day, each up to 2048 tokens. Prediction of protein interactions with other proteins and biomolecules is the main focus of this review. However, regardless of whether the prediction is a single protein sequence or multiple biomolecules the predicted models must be examined to ensure they are reasonable which we discuss in the following section.

## 2. Confidence metrics

Quantifying improvements in the accuracy of protein structure prediction algorithms requires metrics to score the similarity between predicted and experimental structures. However, it is well established that single metrics often prove inadequate [60]. Useful predictions may show high structural similarity over a small proportion of the sequence, or lower similarity but over a larger proportion of the sequence [61]. Additionally, many metrics rely on superimposing predicted and experimental structures. As proteins are often modular and exhibit flexibility between domains, optimal superimposition over one domain may be suboptimal for others, lowering the overall accuracy even if the predictions of the individual domains themselves are highly accurate [62]. Consequently, CASP competitions have used a variety of metrics to score predictions to quantify both global and local accuracy. Neural networks trained to predict protein structures are also trained to estimate the accuracy of the predicted models [63]. AlphaFold generates confidence values for each model as well as metrics defined at the residue level. Researchers should consider all of these metrics when evaluating predicted models (table 2).

**Table 2.** Confidence metrics typically used to assess usefulness of AlphaFold predictions.

metric	model or residue level	description	assessment
pLDDT	residue	confidence of residue modelling	0−100 >90—sidechains correct >70—backbone is correct <50—probably unstructured
PAE	residue	confidence in distance between two residues	0−30 Å (lower→more confident)
pTM	model	confidence of protein fold	0−1 >0.7–0.9 chance of being the correct fold >0.5—likely the correct fold <0.2—unlikely to be correctly folded
replicates	both	repeated computations of the predicted model (typically 1−5)	replicates should superimpose
chemistry	both	is the region chemically sensible?	hydrogen bonding, electrostatic interactions, clash scores, etc.

### 2.1. Predicted local distance difference test

The local distance difference test (LDDT) score was introduced in CASP9 [64], and provides an alignment free, per-residue score of how well local atomic geometry is preserved when a model is compared with a reference structure. The test examines pairs of atoms within a 15 Å radius in the reference structure and determines if their distances are maintained in the model structure within specific tolerance thresholds (0.5, 1, 2 and 4 Å). A final LDDT score is calculated as the average fraction of preserved distances across all four thresholds for atom pairs within the 15 Å radius. The LDDT ranges from 0 to 100, where 100 indicates perfect agreement. AlphaFold generates predicted LDDT (pLDDT) scores as a confidence measure, effectively estimating the reliability of each part of the predicted structure. AF2 considers only Cα carbon atom distances and therefore has a single pLDDT per residue, while AF3 considers all atoms. Of note, pLDDT scores also provide a useful guide for intrinsically disordered regions in proteins, with low scores (<50) correlating with disordered regions. However, it is important to recognize the atomic structure of the low-confidence region will not be accurately modelled [63].

### 2.2. Predicted Aligned Error

**Figure 3.**
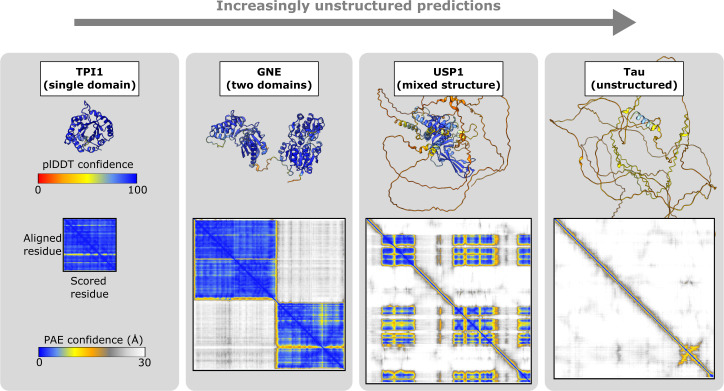
Selected examples of protein monomer predictions and their residue-level confidence scores. Predicted structures are coloured by pLDDT score with PAE plots shown below each structure (AF-P60174-F1-v4, AF-Q9Y223-F1-v4, AF-O94782-F1-v4, AF-P10636-F1-v4).

**Figure 4.**
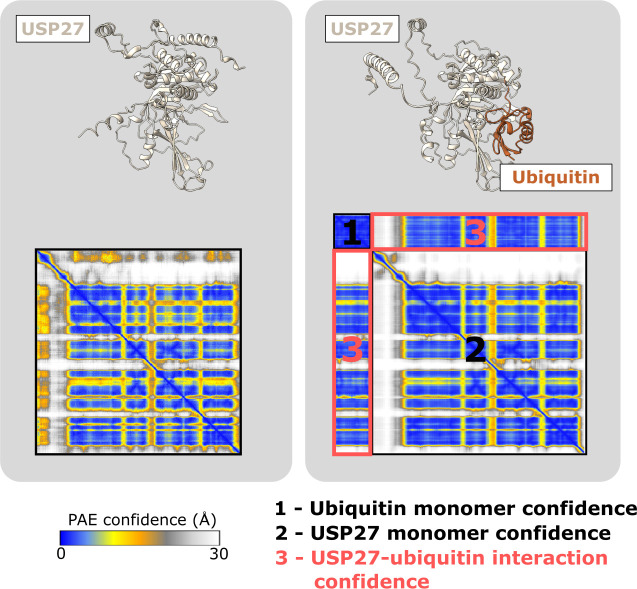
Comparison of a protein monomer versus a PPI prediction. The USP27 monomer (AF-A6NNY8-F1-v4; left) and a prediction of USP27 with ubiquitin (right) [65] are shown. Predicted structures are coloured by polypeptide chain, with PAE plots shown below each structure. The USP27-ubiquitin PAE can be separated into four sections. The two off-diagonal sections (numbered 3) represent the confidence in the interaction.

AlphaFold also generates a score for each residue pair within the prediction that reflects the uncertainty in their positioning relative to each other. All the pairings taken together yield a 2D Predicted Aligned Error (PAE) plot. The PAE scores effectively represent the predicted positional error, in angstroms, between the given model and a hypothetical ground truth structure at residue *i*, if the two structures were superimposed on residue *j*. Examples of PAE plots for various levels of predicted structuredness are shown in figure 3. For well-defined domains, each residue within the domain is confident with respect to the others, producing characteristic squares of high confidence on the main diagonal of the plot (figure 3, left two examples). On the other hand, for disordered proteins only residues adjacent to each other in the amino acid sequence are high confidence, producing a line of confidence along the diagonal (figure 3, rightmost example). For predictions of PPIs, the inter-protein confidence scores are important, which are present on the off-diagonal of the PAE plots [53] (figure 4).

### 2.3. Predicted Template Modelling score

Prior to introduction of the Template Modelling (TM) score, the root mean square deviation (RMSD) was used in CASP. RMSD is calculated from two optimally superimposed atomic structures by taking the square root of the mean of the squared distances between corresponding atoms in the two structures [66]. This measure typically considers only Cα carbon atoms from each residue in the superimposed structures and approaches zero for two identical structures. One shortcoming of using RMSD alone is that a small number of large deviations between the predicted and actual structures can greatly increase the score [67]. This led to the introduction of the TM score in CASP5 [61], in which the deviation between Cα atom positions appears in the denominator of the equation, and is normalized by the length of the protein. Values range between 0 and 1, with unrelated proteins producing a score of approximately 0.2, while scores above 0.7 represent a 90% probability the proteins have the same fold [68].

The predicted TM (pTM) score is calculated by approximating the deviation between residues in the predicted structure and corresponding residues in the hypothetical ground-truth structure, instead of using the actual deviation observed in superimposed structures [37]. For PPIs, the interface pTM (ipTM) score specifically evaluates the confidence of predicted interfaces by considering pairs of residues that lie on different protein chains [53]. It should be noted the pTM and ipTM scores are calculated from the predicted positional error probabilities used to calculate PAE values.

### 2.4. Additional measures

In addition to the confidence metrics provided by AlphaFold, several other criteria can be used. For example, AlphaFold incorporates some diversity in the predicted structures through its ensemble of five neural networks trained with different random seeds and varying template usage. The RMSD of these computational replicates can be used to provide additional assessment of the confidence either at a local level, e.g. between domains, or between the entire models.

The plausibility of the predicted chemical interactions in the region of interest should also be assessed. Experimental structure validation tools such as MolProbity [69] can be used to give additional model-level metrics of prediction quality, and specify regions where the predicted model deviates from ideal bond geometry or introduces clashes between residues.

**Figure 5.**
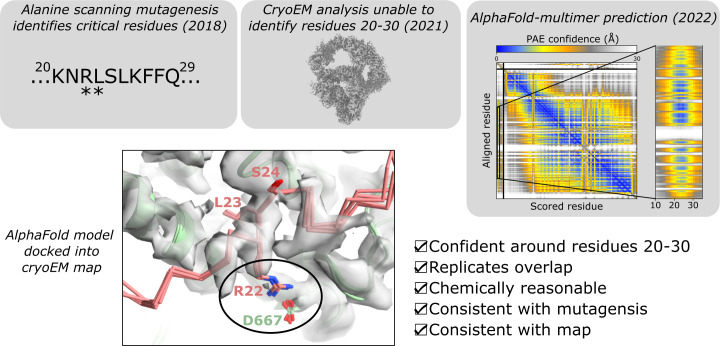
Experimental support for AlphaFold models of the USP1-FANCD2 protein–protein interaction. Critical residues identified by alanine scanning mutagenesis are indicated with asterisks. The inset of the PAE focuses on the region around residues 20−30. The circled region of the AlphaFold models indicates the ionic interaction. Adapted from [70].

Further support for predicted structures comes from experimentally testing mutations designed based on the predictions. This can also be considered retrospectively. For example, alanine scanning mutagenesis had identified key amino acid residues within a disordered region as critical for a protein–protein interaction (figure 5) [71]. Despite experimental structure determination of the assembly using cryogenic electron microscopy (cryoEM), a reliable model of this region was not able to be constructed [72]. AlphaFold predictions between the two proteins revealed a confident interaction in terms of pLDDT and PAE scores. The predictions were chemically sensible, with a leucine on one protein buried into a hydrophobic pocket of the other, and an adjacent arginine forming an ionic interaction with an aspartate [70]. Strikingly, these two residues were the most critical in the original alanine scanning mutagenesis. Subsequent experimental support for the AlphaFold mode of interaction was obtained by focusing on this region in cryoEM maps [70]. Overall, these data convincingly supported the AlphaFold model (figure 5).

### 2.5. Guidance on AlphaFold confidence metrics

While model-level metrics can be used to assess the accuracy of the overall fold, residue-level metrics are typically more useful in experimental design and for flexible and multi-domain proteins. For example, drug design and screening require high confidence in the positioning of active site residues [73], while assessing PPI predictions requires consideration of confidence levels in the residues constituting the interface itself [53,74]. The importance of assessing confidence metrics is exemplified in AF3 where extended disordered regions for large proteins can form bundles of helices [57]. These hallucinations may appear to have reasonable folds but their pLDDT scores are low and likely result from the diffusion-based architecture of AF3.

Any publication reporting AlphaFold predictions that are not available in any database should provide:

(1) pLDDT and PAE confidence metrics
(2) Model files
(3) Details of the prediction methods

At a minimum, the confidence metrics should be illustrated in a figure; however, making the associated files available is preferable. The pLDDT and PAE metrics are generated as json or pickle files, while predicted structures are generated as PDB or CIF files, with the pLDDT scores stored in the B-factor column. ModelArchive is a useful repository that allows upload of predicted structures and associated data (https://modelarchive.org/). Details of the prediction methods should be included in the publication, and can also be included in ModelArchive, for example, the version of the structure prediction software, which templates were used, and whether Amber relaxation of the model was performed. Although it is not common to include the MSA, this may be useful to compare predictions of the same target that use different MSAs, particularly as sequencing databases change. Considering the correlation between MSA depth and prediction quality, reporting the MSA depth may also be useful to consider. Together this information will provide readers with all the information to critique the predictions.

## 3. Classes of protein structure prediction

The uses of AlphaFold have rapidly extended far beyond its initial target of single conformations of native, monomeric proteins. In this section, we discuss these advancements, focusing on how robust and widely applicable they are, and where further improvements may arise. We begin with extensions still involving protein monomers: prediction of conformational changes and mutant proteins. We then consider predictions involving multiple components: protein–protein, protein–nucleic acid and protein–small molecules.

### 3.1. Prediction of multiple conformations

Proteins are often structurally heterogeneous with conformational changes coupled to function, for example, binding of a ligand or phosphorylation of residues can trigger large conformational changes. There is a continuum of structural heterogeneity. At one extreme are intrinsically disordered regions, such as short linear motifs, which can adopt numerous conformational states. However, there are frequently only two dominant states relating to protein function, for example, open versus closed states of ion channels, auto-inhibited versus activated state of enzymes and pre- and post-fusion conformations of viral fusion proteins. Thermodynamic modelling of proteins as two different functional states can also be applied to intrinsically disordered regions [75]. Prediction of such conformational ensembles was first introduced as a category in CASP15 [76].

**Figure 6.**
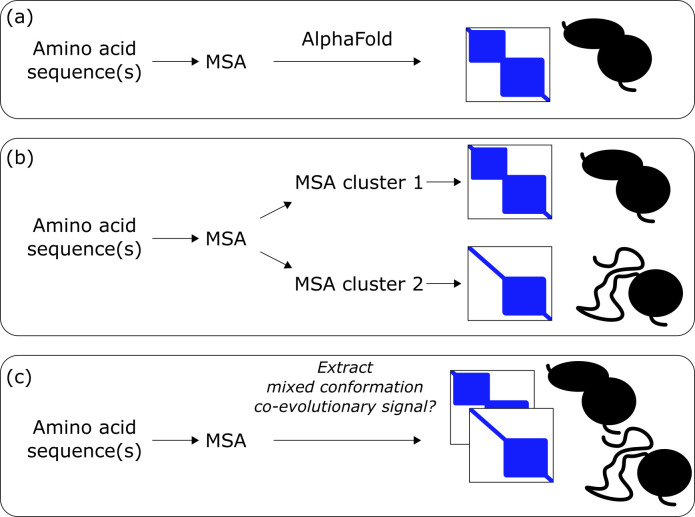
Schematic representation of protein structure prediction for conformational changes. (a) The standard AlphaFold approach favours a single structure per run. (b) Current AlphaFold-based methods for conformational ensembles use sequence clustering or other manipulations of the MSA (middle). (c) A more elegant approach may be to simultaneously generate multiple structures.

AlphaFold has been engineered to predict a single structure, and while it will generally produce a correct conformation, this may be biased towards one state (figure 6a) [77–79]. The balance between number of experimental structures of each conformation in the PDB has shown to be important in determining which conformation AlphaFold favours [79]. However, several approaches have been developed to extract multiple conformations using AlphaFold.

These approaches have typically focused on manipulation of MSAs [77,78,80–84]. Reducing the number of residues considered in the MSA by masking columns [80] and limiting depth of the MSA by sub-sampling sequences have both been explored [77,81–83]. AF-Cluster groups the sequences of the MSA into subsets to explore predicted conformational space [82] (figure 6b); however, random sequence sampling can also be used [77]. The use of template structures can also influence predicted conformations, particularly in cases with shallow MSAs [77]. Random perturbation when the models are generated has also been performed by dropout of layers of the neural network and can increase diversity in the predictions [85]. An adaptation of AF2, Cfold, has been trained specifically on one set of conformational states with other sets being used for evaluation, and with MSA clustering and dropout of layers to search for different conformations [84]. This structure prediction tool was able to predict some conformational changes outside of the training set but struggled with large conformational changes. Another study suggests that conformational changes may only be able to be robustly generated when the training set contains similar-sized clusters of each conformation [79]. In general, there is some disagreement about the impact of co-evolutionary information versus the training set in predicting alternative conformations [83,86–88] and the wider applicability of these approaches remains to be shown.

A more natural approach for protein conformations might be to simultaneously generate two or more predictions from one run (figure 6c). It has been shown that MSAs contain information on protein conformations, where predicted distances between flexible residue pairs tend to have more complex probability landscapes than rigid pairs [89,90]. Deriving multiple conformations simultaneously could avoid the need to cluster sequences into subsets and instead use information from all sequences in modelling all conformations. However, this is complicated by the possible presence of structural variation between homologues that can also imprint on MSAs [91]. These would need to be detangled from true conformational changes. Furthermore, training on structures with multiple conformations may be required [89] as well as more experimental structures covering multiple conformations. MultiSFold has been developed with this approach and provides improvements over AlphaFold2, particularly for cases with lower AlphaFold2 confidence metrics [92]. We expect the prediction of conformational changes to advance rapidly in the coming years and become of general utility.

### 3.2. Prediction of mutants and variants

Point mutations in protein structures are of interest clinically, underlying many genetic diseases and present in cancers. Mutations may cause disease by interfering with enzyme-substrate binding or catalytic mechanisms, which may be evident from the structure alone, or through reducing protein stability. It has been shown that changes in pLDDT confidence scores from AF2 predictions before and after point mutation do not correlate with experimental measurements of protein stability [93]. This is perhaps unsurprising as point mutations will have only a minor effect on the MSA and therefore co-evolutionary information will likely constrain the residue–residue contacts to the wild-type structure. However, the DeepMind team have adapted the AlphaFold system to focus on pathogenicity of point mutations [94]. This involved fine-tuning on variants that were common in humans and primates and assigned to be benign and variants that were absent assigned to be pathogenic. Importantly, AlphaMissense does not predict structural changes due to mutations. However, this is a useful tool when considering the disease relevance of point mutants. Splice variants are another biologically relevant sequence-driven variation in protein structure. However, co-evolutionary information drawn from both canonical and splice variations may confound the structure prediction.

Nevertheless, knowledge of the wild-type protein structure can be used to design precise mutations that disrupt specific aspects of a proteins function. In the absence of structural information, a simple approach is to perform a series of truncations to identify important regions for a particular function. However, many proteins fold back upon themselves creating complex relationships between sequence and structure meaning that many truncations may extensively disrupt the protein structure. With predicted structures of proteins and their complexes readily available, there is no place for ‘blind’ truncations and instead experimental or predicted structures should be used to inform design of mutations to examine protein function.

AlphaFold predictions also aid in design of variants for recombinant protein expression in functional studies and biotechnology. Recombinantly expressed proteins are often insoluble, and the truncation of proteins is commonly used to improve solubility. Having more accurate predicted structures allows for more efficient selection of soluble protein domains for expression in the absence of experimental structures.

Proteins are frequently engineered with tags such as antigens or fluorescent proteins to facilitate cellular experiments or protein purification. However, such tags can affect protein function [95,96]. This is particularly so if the residue(s) adjacent to the tag fusion site are structurally important. Examination of AlphaFold predictions at the tag site may be useful in predicting if the tag will disrupt function. Predictions of tagged variants may also be useful in identifying and circumventing these limitations; however, these predictions will likely also suffer from the limitations described above as the sequences are engineered and so co-evolutionary information will be confounded. In either case predictions of tagged variants are useful in visualizing the size of the tag compared with the protein of interest and provide structural context at the fusion site.

### 3.3. Prediction of protein–protein interactions

AlphaFold predictions of assemblies between multiple proteins can be used for various purposes: to identify novel interacting partners, provide atomic models of well-characterized interactions that have proven intractable to experimental determination, and to resolve ambiguities in experimentally determined maps of protein assemblies. However, creating databases of predicted protein assemblies is much more computationally demanding than for monomer databases. In the human proteome, there are over 200 million possible pairwise combinations of proteins, let alone higher order assemblies. Nevertheless, organisms with simpler proteomes, such as yeast, have been studied from this perspective [97] and a dedicated deep-learning network for rapid prediction of PPI models has been applied to the human proteome [47]. There have also been several efforts targeting subsets of proteomes: (i) experimentally supported PPIs from the human proteome have been used to guide prediction of binary and higher order protein assemblies [98,99], (ii) signalling and metabolic pathways have been used to focus the predictions [48,100,101], (iii) cross-kingdom plant–pathogen interactions have been screened [102], (iv) protein ligands and peptides have been screened against protein receptors to predict the native pairings [103–105], and (v) protein functions such as genome maintenance have been used to screen for pairwise protein–protein interactors [46,106]. Several databases for these ‘predictomes’ have been constructed (table 1).

**Figure 7.**
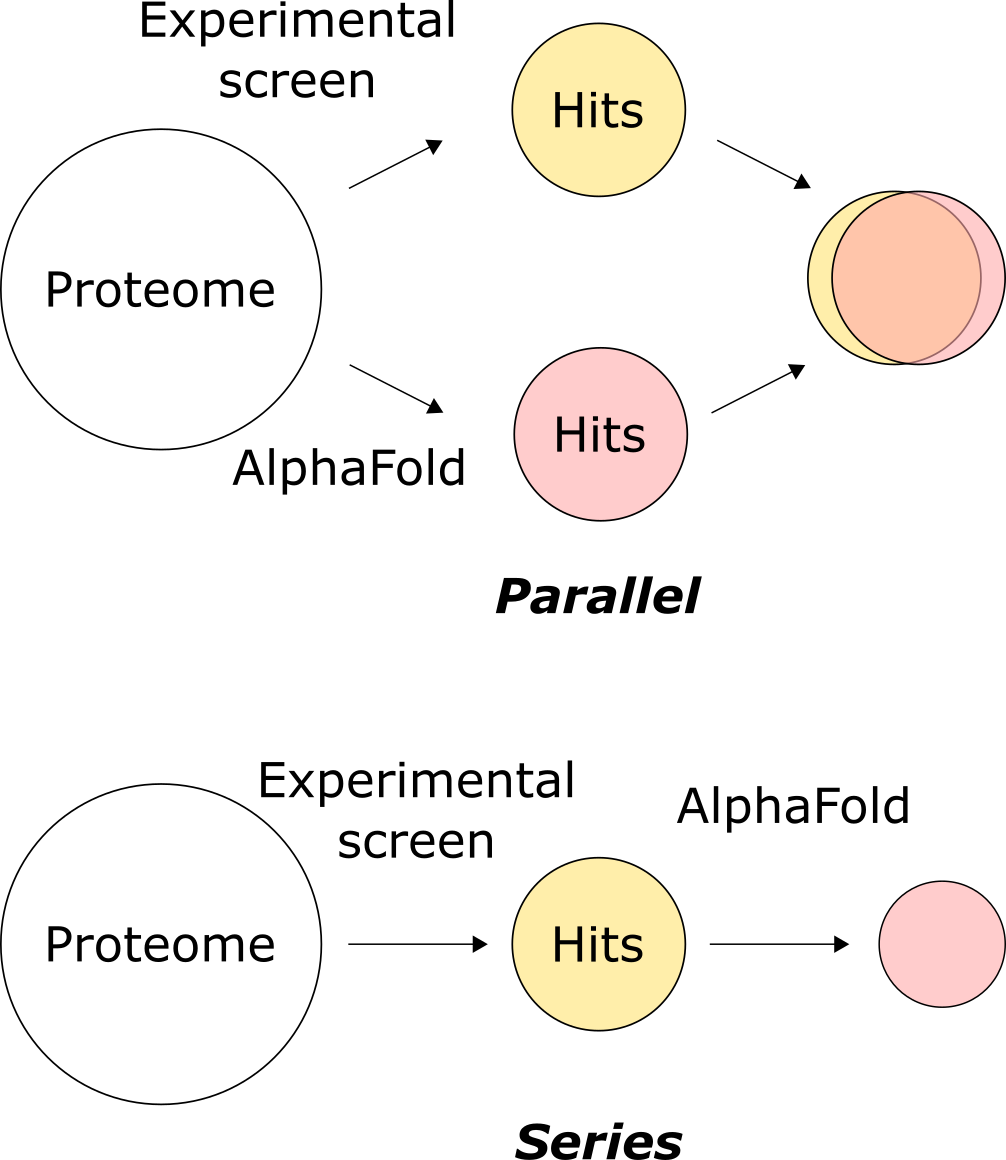
Alternative approaches to incorporating structure prediction into high-throughput screening of PPIs.

Protein structure prediction can also be integrated into experimental pipelines. High-throughput identification of interacting proteins can be achieved experimentally by various methods, including crosslinking mass spectrometry, genome-wide association studies, yeast two-hybrid and protein microarrays. Predictions of protein–protein assemblies provide a computational high-throughput approach that can complement these experimental techniques (figure 7). This can be performed in parallel, where interactions of a protein of interest with all other proteins in the proteome are probed through the experimental technique and predictions, respectively. The overlap between the sets of identified interactors are then considered as the most confidently assigned interactors. Alternatively, predictions could be used in series, following high-throughput experimental techniques, to reduce the number of false-positive interactions. This would require only computing protein–protein pairs that are supported by the high-throughput technique and so is less computationally expensive than the former but may miss some interactors that were not experimentally detected. For both of these approaches, a bait protein is screened against a list of potential interactors. AlphaPulldown software based on AF2-multimer has been developed for this ‘one-vs-all’ purpose [74]. Furthermore, AlphaLink, also derived from AF2, and Chai-1 software allow incorporation of distance constraints between atoms into structure predictions (e.g. from crosslinking experiments) [59,107].

In prediction screening studies, it is critical to minimize false-positive interactors, which raises an important question—where do we draw the line between an interaction or non-interaction? Negative controls for prediction screening should be considered, for example, a scrambled sequence of the target. Thresholds derived from global confidence metrics such as the ipTM can also be useful in some cases. However, many proteins interact via short motifs which can be hard to pick out in large proteins and it has been demonstrated that individually, AlphaFold metrics are not very useful in large screening approaches [46,48]. pLDDT and PAE scores considering only interface residues provide an improvement over the average pLDDT and pTM scores [108]. Another simple approach derived from the PAE has been proposed [48]. This metric uses areas of the PAE involving inter-protein residue pairs, i.e. the contact PAE scores, focusing on those below a defined threshold (12 Å was found to be best). Similar to this approach, AF3 provides a minimum inter-protein PAE score. A metric that considers the agreement in predicted contacts between replicate models has also been developed, known as the ‘average n models’ [106]. More complex assessments involving combinations of metrics have also been developed. In particular, it has been found that incorporating additional biological metrics was superior to just using structure-based metrics such as the PAE [46]. This combined metric is known as the Structure Prediction and Omics informed Classifier (SPOC) and is available through the predictomes website (table 1). These various metrics allow ranking of predictions; however, caution should be taken with high-throughput screening against paralogues as these may generate confident predictions for non-interactors [105].

In addition to high-throughput screening approaches, predictions can also inform more focused mechanistic studies. Integrative structural biology combines multiple methods to determine structural features of difficult targets, such as huge protein assemblies like the nuclear pore complex [109]. AlphaFold models have been combined with cryoEM to model the nuclear pore complex [110,111]. The wealth of atomic models of PPIs derived from predictions now means experimental validation and mechanistic studies of clusters of PPIs are a bottleneck.

### 3.4. Prediction of protein binders

Design of novel proteins that bind to natural proteins is an ambitious goal that has applications, including antibody replacements, biosensors and tools that disrupt protein interactions within cells. Several new competitions have arisen to independently test designed proteins and binders [112]. While experimental methods, such as directed evolution, have been used in protein design, they are laborious and computational design has the potential to significantly reduce the experimental burden. State-of-the-art structure prediction has provided significant boosts to computational success rates [113–115]. There is particular emphasis on the design of protein binders that allow targeting of a particular region on a target protein [113,116–118]. There have also been adaptions for binder interfaces involving β-strands [119], binders of α-helices [120] and design of cyclic peptides [121–123]. Further extensions may look to the incorporation of non-natural amino acids.

These methods for binder design rely on computing thousands of different designs, both in terms of backbone geometry and sequence, then filtering them to a limited number for experimental validation of binding. Backbone geometry has been designed using either hallucination or diffusion deep-learning approaches [113,117], while a graph neural network architecture, ProteinMPNN, has been used to design the sequences [124,125]. The requirement to screen thousands of designs *in silico* makes these methods computationally expensive; however, a reduction in numbers needed to be experimental tested offsets this cost. Although success rates vary significantly between targets, the methods are now at a level where fewer than 100 designs may need to be experimentally tested to obtain binders (table 3). BindCraft is particularly promising with success rates of 10–100% across 12 different protein targets, and yielding the best *de novo* binders in a recent competition by Adaptyv Bio [126]. However, there are still challenging targets that have proved intractable [118] and will likely require testing of an order of magnitude more designs and/or advances at the computational level.

**Table 3.** Computational tools developed for protein binder design.

software	experimental success (%)^a^	predictions experimentally tested per target	code licence
RFdiffusion [113]	7−35 (5 targets)	<100	BSD License
BindCraft [117]	10−100 (12 targets)	6−53	MIT License
AlphaProteo [118]	9−88 (7 targets) 0−88 (8 targets)^b^	47−172	not currently available
RFpeptides [123]	21−38	6−14^c^	code to be released

^a^ Designs binding/designs experimentally tested, each publication used different binding targets and different criteria for success so this is only a rough comparison.

^b^ TNFα subsequently added as a hard target.

^c^ Additional designs failed at the chemical synthesis step.

One major class of hard targets is short linear motifs. These are short regions of proteins, generally 3−15 amino acids long, which participate in PPIs. Although their small size increases the computational tractability, this is offset by the fact they are typically disordered in the absence of their binding partner. Designed binders for these targets could allow for more targeted modulation of PPIs. In applications of designed binders in a biological context, determination of specificity and selectivity will be critical.

Antibodies are natural protein binders that specifically recognize antigens. Antibody–antigen protein pairs are not as reliably predicted as protein–protein interactions in general. This is unsurprising since co-evolutionary information is not necessarily present in the binding regions of antibodies. AF3 appears to have narrowed the gap between prediction accuracy for antibody–antigen and general protein–protein pairs; however, may require generation of hundreds to thousands of predictions with different seeding [53,57]. Similarly, AFsample and MassiveFold, both based on AF2-multimer and using thousands of predictions, were able to generate some high-confidence antibody–antigen predictions [85,127].

Given the importance of co-evolutionary information in determining accurate structures, one may ask: how is this information incorporated into binder design and antibody–antigen predictions? Binders are typically designed to be very small, so it may not be unreasonable to expect it is only the learned structural information that is dominating the usefulness for these designs. However, another possibility is that these tools are essentially very complex ‘structure mixers’ collecting mini-motifs from many different proteins and assembling them into a single protein. Regardless, it is clear that computational protein design is rapidly maturing owing to the advances in deep-learning.

### 3.5. Prediction of protein–nucleic acid and protein–small molecule interactions

Proteins interact with nucleic acids during a variety of cellular processes, and so protein–nucleic acid assemblies are another important target for structural biology. As with conformational ensembles, RNA and protein-RNA assemblies were introduced in CASP15 [128] and DNA included in the recent CASP16 competition. The next-generation structure prediction tools, AF3, RosettaFold-All-Atom, Chai-1 and Boltz-1 allow input of arbitrary nucleic acid sequences. Although AF3 is state of the art with respect to nucleic acids, it has not reached the same level of general applicability as AF2/AF2-multimer achieved for protein monomers and PPIs [129]. There are far fewer experimental structures of protein–nucleic acids than protein monomers or protein–protein assemblies for deep-learning tools to draw information from, and so further experimental structures of protein–nucleic acid assemblies would be useful to advance this class of protein structure prediction.

Proteins can also bind to small molecule ligands as part of their biological function or when targeted with small molecule drugs. Prediction of the latter is important for drug development and is seeing considerable interest. While there are a large number of protein–small molecule structures in the PDB, there are additional complexities when considering a prediction tool that can cover all possible small molecules compared with the protein-only classes discussed in the earlier sections.

**Figure 8.**
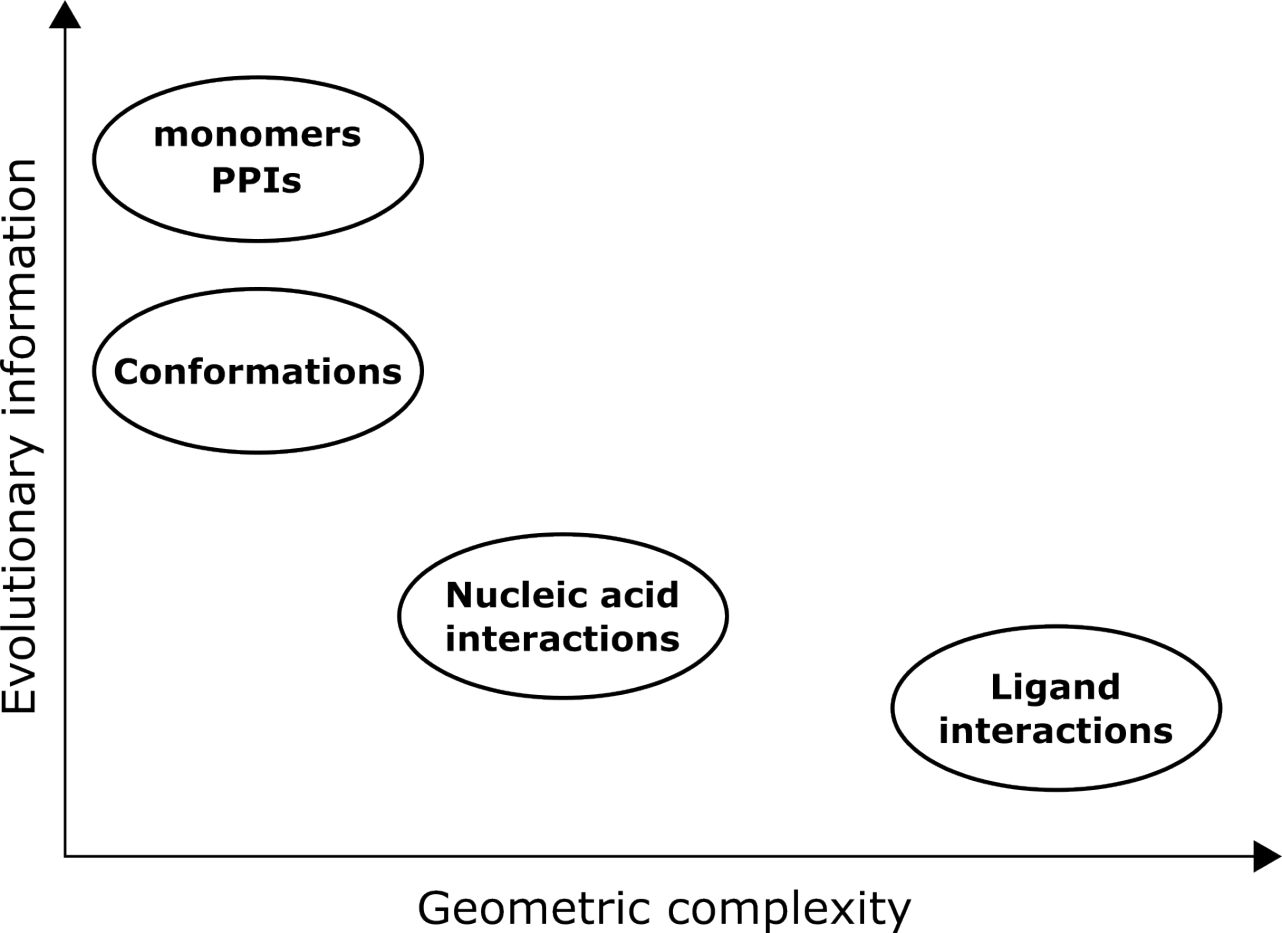
Comparison of the difficulty of general solutions for classes of protein structure prediction. Co-evolutionary information refers to that which can be extracted from MSAs, while chemical complexity refers to the number of possible atom types and functional groups in the range of possible structures to be predicted.

We consider prediction complexity of a class of targets to depend on the chemical complexity of all possible molecules relating to the class, and the potential co-evolutionary information that can be extracted (figure 8). While size of the prediction target is also important, protein and nucleic acid polymers have restricted geometry that limits the complexity added due to size, for example, protein polymers have limited backbone angles that are energetically favourable as observed in the Ramachandran plot.

Chemical complexity considers the number of functional groups and atom types. For polymers, chemical complexity is related to the number of different types of building blocks: 20 for proteins, four for DNAs and four for RNAs. Furthermore, nucleic acids typically form oligomers mediated by base pairing. While double-stranded DNA is the primary example, there are many other complex nucleic acid assemblies that are biologically important, such as Holliday junctions, D-loops and R-loops. As such, prediction of arbitrary protein–nucleic acid assemblies is more chemically complex than any of the protein-only classes. Furthermore, small molecules generally lack the regular structure of polymers, although small molecules are much smaller than proteins. Importantly, predictions for the protein-small molecule class may require modelling of various functional groups and atom types not seen in proteins or nucleic acids. Small molecules may even consist of modified amino acid or nucleic acid units. Therefore, a tool that reliably predicts arbitrary protein–small molecule assemblies has to account for the highest level of chemical complexity.

Co-evolutionary information for prediction in the protein–nucleic acid and protein–small molecule classes is not available to the same extent as for protein–protein assemblies. For the protein–small molecule class, co-evolutionary information is essentially one-sided, i.e. protein residues at the binding site may be conserved but there is not additional information from MSAs involving the interacting partner to signal co-evolution, unlike for PPIs. Furthermore, small molecule binding can drive reordering of the hydrophobic core of a protein structure [70] and if such sites do not have a natural ligand, this could even conflict with co-evolutionary information.

Genomic DNA sequences clearly evolve, raising the question: is there useful co-evolutionary information that can be extracted for prediction of protein–nucleic acid complexes? The same is true for RNA sequences. While there are large databases of DNA and RNA sequences, there are only four nucleotide units each for DNA and RNA compared with the 20 amino acids of proteins. Nevertheless, co-evolutionary information has been extracted from MSAs of RNA sequences and used for prediction of RNA-only structures [130]. Furthermore, there are cases where proteins recognize specific nucleic acid sequences, for example, transcription factors [131], suggesting there may be evolutionary coupling between the protein sequence and nucleic acid sequence. In such cases, hybrid MSAs could be generated with each amino acid sequence paired with a nucleotide sequence from the same species. Overall, there is more potential for co-evolutionary information in protein interaction with nucleic acids than for small molecules; however, whether this is useful in the prediction of such assemblies remains to be seen.

Given the high chemical complexity and reduced co-evolutionary information for protein–small molecules, we expect multiple major technical breakthroughs will be required before generally applicable structure prediction of this class will be solved. However, a subset of the problem that is relevant for drug design may be achievable with current technology—when a small molecule binding site is already known and new small molecules are to be modelled in place. This could be particularly useful when coupled to prediction for paralogues to check for off target binding and reliable prediction of small molecule binding affinity.

## 4. Outlook

‘All models are wrong but some are useful’ is a saying in statistics attributed to George Box. It is abundantly clear that the current state-of-the-art structure prediction tools have widespread use for studying both single protein chains and protein–protein assemblies. The predictions serve as powerful hypothesis generators for experimental validation and this is now a bottleneck in many aspects of biology. Moreover, they contain experimental data embedded within the predictions in the form of protein co-evolution derived from massive sequencing efforts. This makes them useful complements to support other experimental techniques, both low- and high-throughput, beyond hypothesis generation. AlphaFold can be particularly useful for PPIs that are difficult to capture experimentally, for example, transient interactions which may still leave their mark on evolutionary couplings. However, it is also important to keep in mind that the usefulness of AlphaFold and other structure prediction tools will always depend on the biological question.

Repurposing of AlphaFold from protein monomers to PPIs came within months of the original release. The next generation of deep-learning tools for structural predictions of interactions with non-proteins have arrived three years after the initial AlphaFold release; however, we do not believe that these have reached general utility yet. Unlike for PPIs, the co-evolutionary signal within MSAs effectively becomes ‘one-sided’ when protein–nucleic acid and protein–small molecule interactions are considered. While template structures can be used to guide structure predictions for protein–nucleic acid and other interactions, their requirement limits generalizability. For protein monomers and PPIs, templates are frequently dispensable with AlphaFold, highlighting the general utility for these prediction classes. More protein–nucleic acid experimental structures, particularly those with different DNA structures and nucleotide sequences, will allow more useful predictions and more rigorous assessment of protein–nucleic acid predictions. However, there will need to be significant technical developments to allow general utility of these tools for broader biomolecular interactions.

Major applications of structure prediction in the immediate future will likely come from design of protein binders and modelling of protein conformational changes. Both of these remain in the realm of protein-only structure; however, will be immensely useful for biological and biotechnological applications. Although protein conformational changes have more complex co-evolutionary signals than rigid proteins, they retain a ‘two-sided’ signal. It is possible that extracting useful structural information about protein conformational changes may not be generalizable. However, deconvoluting direct from indirect evolutionary couplings yielded improved performance of the MSA approach, and it is tempting to speculate—could deconvoluting the signal from multiple conformations boost the accuracy of all protein structure predictions even further?

Prediction of large structures remains a computational challenge. Longer sequences require more GPU memory at inference time and even AF3 is capped at 5120 amino acid residues [57]. Furthermore, elongated structures such as coiled coils can become spuriously bent [132], while in AF3, disordered regions in large structures have a tendency to collapse into helical bundles. Large disordered regions can also impede identification of PPIs [133]. Predictions using truncations can get around some of these issues. Significant updates in speed of generating predictions have come from faster MSA generation, with a GPU implementation recently released [134]. This will facilitate more high-throughput predictome approaches.

Just as the expansion of gene databases enabled the dramatic improvement in protein structural prediction, improvements in structural prediction can improve the quality of gene databases and provide novel evolutionary insights. As proteins are more conserved at the structural level than sequence level, alignment and clustering of predicted structures allows for the identification of homologues that would not be detected by sequence-based methods. This has allowed for improved annotation of evolutionarily distant but conserved proteins and identification of novel protein folds [135]. Such an approach allows for greater insight into evolutionary history across all kingdoms of life and viruses, through construction of structure-based phylogenetic trees and identification of horizontal gene transfer events that often accompany host–parasite co-evolution [41].

Overall, AlphaFold has led to a massive wealth of structural information in prediction databases and bespoke applications of the tool. Local confidence metrics, self-reported by the AlphaFold network for every prediction, facilitate determining the usefulness of each prediction. Yet, the atomic models still need to be related to protein function and so in our opinion the structure prediction revolution has, if anything, increased the need for chemical and structural literacy amongst biologists.

## Data Availability

This article has no additional data.

## References

[1] Kendrew JC, Bodo G, Dintzis HM, Parrish RG, Wyckoff H, Phillips DC. 1958 A three-dimensional model of the myoglobin molecule obtained by X-ray analysis. Nature **181**, 662–666. (10.1038/181662a0) 13517261

[2] Grabowski M, Niedzialkowska E, Zimmerman MD, Minor W. 2016 The impact of structural genomics: the first quindecennial. J. Struct. Funct. Genom. **17**, 1–16. (10.1007/s10969-016-9201-5) PMC4834271 26935210

[3] Levinthal C. 1969 How to fold graciously. Mössbauer Spectrosc. Biol. Syst. Proc. **24**, 22–24.

[4] Kerner MJ *et al*. 2005 Proteome-wide analysis of chaperonin-dependent protein folding in Escherichia coli. Cell **122**, 209–220. (10.1016/j.cell.2005.05.028) 16051146

[5] Ciryam P, Morimoto RI, Vendruscolo M, Dobson CM, O’Brien EP. 2013 In vivo translation rates can substantially delay the cotranslational folding of the Escherichia coli cytosolic proteome. Proc. Natl Acad. Sci. USA **110**, E132–40. (10.1073/pnas.1213624110) 23256155 PMC3545769

[6] Waudby CA, Dobson CM, Christodoulou J. 2019 Nature and regulation of protein folding on the ribosome. Trends Biochem. Sci. **44**, 914–926. (10.1016/j.tibs.2019.06.008) 31301980 PMC7471843

[7] Dill KA, Ozkan SB, Shell MS, Weikl TR. 2008 The protein folding problem. Annu. Rev. Biophys. **37**, 289–316. (10.1146/annurev.biophys.37.092707.153558) 18573083 PMC2443096

[8] Moult J, Pedersen JT, Judson R, Fidelis K. 1995 A large-scale experiment to assess protein structure prediction methods. Proteins **23**, ii–v. (10.1002/prot.340230303) 8710822

[9] Varadi M *et al*. 2024 AlphaFold protein structure database in 2024: providing structure coverage for over 214 million protein sequences. Nucleic Acids Res. **52**, D368–D375. (10.1093/nar/gkad1011) 37933859 PMC10767828

[10] Altschuh D, Lesk AM, Bloomer AC, Klug A. 1987 Correlation of co-ordinated amino acid substitutions with function in viruses related to tobacco mosaic virus. J. Mol. Biol. **193**, 693–707. (10.1016/0022-2836(87)90352-4) 3612789

[11] Göbel U, Sander C, Schneider R, Valencia A. 1994 Correlated mutations and residue contacts in proteins. Proteins **18**, 309–317. (10.1002/prot.340180402) 8208723

[12] Neher E. 1994 How frequent are correlated changes in families of protein sequences? Proc. Natl Acad. Sci. USA **91**, 98–102. (10.1073/pnas.91.1.98) 8278414 PMC42893

[13] Monastyrskyy B, D’Andrea D, Fidelis K, Tramontano A, Kryshtafovych A. 2014 Evaluation of residue-residue contact prediction in CASP10. Proteins **82**, 138–153. (10.1002/prot.24340) 23760879 PMC3823628

[14] Burger L, van Nimwegen E. 2010 Disentangling direct from indirect co-evolution of residues in protein alignments. PLoS Comput. Biol. **6**, e1000633. (10.1371/journal.pcbi.1000633) 20052271 PMC2793430

[15] Marks DS, Colwell LJ, Sheridan R, Hopf TA, Pagnani A, Zecchina R, Sander C. 2011 Protein 3D structure computed from evolutionary sequence variation. PLoS One **6**, e28766. (10.1371/journal.pone.0028766) 22163331 PMC3233603

[16] Morcos F *et al*. 2011 Direct-coupling analysis of residue coevolution captures native contacts across many protein families. Proc. Natl Acad. Sci. USA **108**, E1293–301. (10.1073/pnas.1111471108) 22106262 PMC3241805

[17] Jones DT, Buchan DWA, Cozzetto D, Pontil M. 2012 PSICOV: precise structural contact prediction using sparse inverse covariance estimation on large multiple sequence alignments. Bioinformatics **28**, 184–190. (10.1093/bioinformatics/btr638) 22101153

[18] Kamisetty H, Ovchinnikov S, Baker D. 2013 Assessing the utility of coevolution-based residue-residue contact predictions in a sequence- and structure-rich era. Proc. Natl Acad. Sci. USA **110**, 15674–15679. (10.1073/pnas.1314045110) 24009338 PMC3785744

[19] Ekeberg M, Lövkvist C, Lan Y, Weigt M, Aurell E. 2013 Improved contact prediction in proteins: using pseudolikelihoods to infer potts models. Phys. Rev. E **87**, 012707. (10.1103/PhysRevE.87.012707) 23410359

[20] Monastyrskyy B, D’Andrea D, Fidelis K, Tramontano A, Kryshtafovych A. 2016 New encouraging developments in contact prediction: assessment of the CASP11 results. Proteins **84**, 131–144. (10.1002/prot.24943) 26474083 PMC4834069

[21] Jones DT, Singh T, Kosciolek T, Tetchner S. 2015 MetaPSICOV: combining coevolution methods for accurate prediction of contacts and long range hydrogen bonding in proteins. Bioinformatics **31**, 999–1006. (10.1093/bioinformatics/btu791) 25431331 PMC4382908

[22] Wang S, Sun S, Li Z, Zhang R, Xu J. 2017 Accurate de novo prediction of protein contact map by ultra-deep learning model. PLoS Comput. Biol. **13**, e1005324. (10.1371/journal.pcbi.1005324) 28056090 PMC5249242

[23] Jones DT, Kandathil SM. 2018 High precision in protein contact prediction using fully convolutional neural networks and minimal sequence features. Bioinformatics **34**, 3308–3315. (10.1093/bioinformatics/bty341) 29718112 PMC6157083

[24] Adhikari B, Hou J, Cheng J. 2018 DNCON2: improved protein contact prediction using two-level deep convolutional neural networks. Bioinformatics **34**, 1466–1472. (10.1093/bioinformatics/btx781) 29228185 PMC5925776

[25] Senior AW *et al*. 2020 Improved protein structure prediction using potentials from deep learning. Nature **577**, 706–710. (10.1038/s41586-019-1923-7) 31942072

[26] Krizhevsky A, Sutskever I, Hinton G. 2012 ImageNet classification with deep convolutional neural networks. Adv. Neural Inf. Process. Syst. **25**, 1097–1105. (10.5555/2999134.2999257)

[27] Vaswani A, Shazeer N, Parmar N, Uszkoreit J, Jones L, Gomez AN, Kaiser Ł, Polosukhin I. 2017 Attention is all you need. Adv. Neural Inf. Process. Syst **30**, 6000–6010. (10.5555/3295222.3295349)

[28] Dayhoff M, Eck RV, Chang M, Sochard M. 1965 Atlas of protein sequence and structure. Silver Spring, MD: National Biomedical Research Foundation.

[29] UniProt Consortium T. 2018 UniProt: the universal protein knowledgebase. Nucleic Acids Res. **46**, 523–531. (10.1093/nar/gky092) PMC5861450 29425356

[30] Mirdita M, von den Driesch L, Galiez C, Martin MJ, Söding J, Steinegger M. 2017 Uniclust databases of clustered and deeply annotated protein sequences and alignments. Nucleic Acids Res. **45**, D170–D176. (10.1093/nar/gkw1081) 27899574 PMC5614098

[31] Richardson L *et al*. 2023 MGnify: the microbiome sequence data analysis resource in 2023. Nucleic Acids Res. **51**, D753–D759. (10.1093/nar/gkac1080) 36477304 PMC9825492

[32] Johnson LS, Eddy SR, Portugaly E. 2010 Hidden Markov model speed heuristic and iterative HMM search procedure. BMC Bioinform. **11**, 431. (10.1186/1471-2105-11-431) PMC2931519 20718988

[33] Remmert M, Biegert A, Hauser A, Söding J. 2012 HHblits: lightning-fast iterative protein sequence searching by HMM-HMM alignment. Nat. Methods **9**, 173–175. (10.1038/nmeth.1818) 22198341

[34] Steinegger M, Söding J. 2017 MMseqs2 enables sensitive protein sequence searching for the analysis of massive data sets. Nat. Biotechnol. **35**, 1026–1028. (10.1038/nbt.3988) 29035372

[35] . Burley SK et al. 2019 Protein Data Bank: the single global archive for 3D macromolecular structure data. Nucleic Acids Res. 47, D520–D528. (10.1093/nar/gky949) 30357364 PMC6324056

[36] Kryshtafovych A, Schwede T, Topf M, Fidelis K, Moult J. 2021 Critical assessment of methods of protein structure prediction (CASP)-round XIV. Proteins **89**, 1607–1617. (10.1002/prot.26237) 34533838 PMC8726744

[37] Jumper J *et al*. 2021 Highly accurate protein structure prediction with alphafold. Nature **596**, 583–589. (10.1038/s41586-021-03819-2) 34265844 PMC8371605

[38] Baek M *et al*. 2021 Accurate prediction of protein structures and interactions using a three-track neural network. Science **373**, 871–876. (10.1126/science.abj8754) 34282049 PMC7612213

[39] Lin Z *et al*. 2023 Evolutionary-scale prediction of atomic-level protein structure with a language model. Science **379**, 1123–1130. (10.1126/science.ade2574) 36927031

[40] Hayes T *et al*. 2025 Simulating 500 million years of evolution with a language model. Science **387**, 850–858. (10.1126/science.ads0018) 39818825

[41] Mifsud JCO, Lytras S, Oliver MR, Toon K, Costa VA, Holmes EC, Grove J. 2024 Mapping glycoprotein structure reveals Flaviviridae evolutionary history. Nature **633**, 695–703. (10.1038/s41586-024-07899-8) 39232167 PMC11410658

[42] Zhang Z, Wayment-Steele HK, Brixi G, Wang H, Kern D, Ovchinnikov S. 2024 Protein language models learn evolutionary statistics of interacting sequence motifs. Proc. Natl Acad. Sci. USA **121**, e2406285121. (10.1073/pnas.2406285121) 39467119 PMC11551344

[43] Litvin U, Lytras S, Jack A, Robertson DL, Grove J, Hughes J. 2024 Viro3D: a comprehensive database of virus protein structure predictions. bioRxiv. (10.1101/2024.12.19.629443) PMC12583693 40958060

[44] Kim RS, Levy Karin E, Mirdita M, Chikhi R, Steinegger M. 2025 BFVD—a large repository of predicted viral protein structures. Nucleic Acids Res. **53**, D340–D347. (10.1093/nar/gkae1119) 39574394 PMC11701548

[45] Nomburg J, Doherty EE, Price N, Bellieny-Rabelo D, Zhu YK, Doudna JA. 2024 Birth of protein folds and functions in the virome. Nature **633**, 710–717. (10.1038/s41586-024-07809-y) 39187718 PMC11410667

[46] Schmid EW, Walter JC. 2025 Predictomes, a classifier-curated database of AlphaFold-modeled protein-protein interactions. Mol. Cell S1097-2765(25)00105-4. (10.1016/j.molcel.2025.01.034) PMC11931459 40015271

[47] Zhang J *et al*. 2024 Computing the Human Interactome. bioRxiv. (10.1101/2024.10.01.615885)

[48] Kim AR, Hu Y, Comjean A, Rodiger J, Mohr SE, Perrimon N. 2024 Enhanced protein-protein interaction discovery via AlphaFold-Multimer. bioRxiv. (10.1101/2024.02.19.580970)

[49] Sánchez Rodríguez F, Chojnowski G, Keegan RM, Rigden DJ. 2022 Using deep-learning predictions of inter-residue distances for model validation. Acta Crystallogr. D Struct. Biol. **78**, 1412–1427. (10.1107/s2059798322010415) 36458613 PMC9716559

[50] Simpkin A, Chojnowski G, Ronan M, Rigden D. 2024 Using deep learning predictions reveals a large number of register errors in PDB. IUCrJ **11**, 938–950. (10.1107/S2052252524009114) PMC11533997 39387575

[51] Oeffner RD, Croll TI, Millán C, Poon BK, Schlicksup CJ, Read RJ, Terwilliger TC. 2022 Putting AlphaFold models to work with phenix.process_predicted_model and ISOLDE. Acta Crystallogr. D Struct. Biol. **78**, 1303–1314. (10.1107/S2059798322010026) 36322415 PMC9629492

[52] McCoy AJ, Sammito MD, Read RJ. 2022 Implications of AlphaFold 2 for crystallographic phasing by molecular replacement. Acta Crystallogr. D Struct. Biol. **78**, 1–13. (10.1107/s2059798321012122) 34981757 PMC8725160

[53] Evans R *et al*. 2022 Protein complex prediction with AlphaFold-Multimer. bioRxiv. (10.1101/2021.10.04.463034)

[54] Bryant P, Pozzati G, Elofsson A. 2022 Improved prediction of protein–protein interactions using AlphaFold2. Nat. Commun. **13**, 1265. (10.1038/s41467-022-28865-w) 35273146 PMC8913741

[55] Mirdita M, Schütze K, Moriwaki Y, Heo L, Ovchinnikov S, Steinegger M. 2022 ColabFold: making protein folding accessible to all. Nat. Methods **19**, 679–682. (10.1038/s41592-022-01488-1) 35637307 PMC9184281

[56] Wohlwend J *et al*. 2024 Boltz-1 democratizing biomolecular interaction modeling. bioRxiv. (10.1101/2024.11.19.624167)

[57] Abramson J *et al*. 2024 Accurate structure prediction of biomolecular interactions with AlphaFold 3. Nature **630**, 493–500. (10.1038/s41586-024-07487-w) 38718835 PMC11168924

[58] Krishna R *et al*. 2024 Generalized biomolecular modeling and design with RoseTTAFold All-Atom. Science **384**, eadl2528. (10.1126/science.adl2528)38452047

[59] Discovery C, Boitreaud J, Dent J, McPartlon M, Meier J, Reis V, Rogozhnikov A, Wu K. 2024 Chai-1: decoding the molecular interactions of life. bioRxiv. (10.1101/2024.10.10.615955)

[60] Moult J, Hubbard T, Bryant SH, Fidelis K, Pedersen JT. 1997 Critical assessment of methods of protein structure prediction (CASP): round II. Proteins **29**, 2–6. 9485489

[61] Zhang Y, Skolnick J. 2004 Scoring function for automated assessment of protein structure template quality. Proteins **57**, 702–710. (10.1002/prot.20264) 15476259

[62] Damm KL, Carlson HA. 2006 Gaussian-weighted RMSD superposition of proteins: a structural comparison for flexible proteins and predicted protein structures. Biophys. J. **90**, 4558–4573. (10.1529/biophysj.105.066654) 16565070 PMC1471868

[63] Ruff KM, Pappu RV. 2021 AlphaFold and implications for intrinsically disordered proteins. J. Mol. Biol. **433**, 167208. (10.1016/j.jmb.2021.167208) 34418423

[64] Mariani V, Biasini M, Barbato A, Schwede T. 2013 lDDT: a local superposition-free score for comparing protein structures and models using distance difference tests. Bioinformatics **29**, 2722–2728. (10.1093/bioinformatics/btt473) 23986568 PMC3799472

[65] Koch I *et al*. 2024 USP27X variants underlying X-linked intellectual disability disrupt protein function via distinct mechanisms. Life Sci. Alliance **7**, e202302258. (10.26508/lsa.202302258) 38182161 PMC10770416

[66] Kabsch W. 1976 A solution for the best rotation to relate two sets of vectors. Acta Crystallogr. A Found. Adv. **32**, 922–923. (10.1107/s0567739476001873)

[67] Kufareva I, Abagyan R. 2012 Methods of protein structure comparison. Methods Mol. Biol. **857**, 231–257. (10.1007/978-1-61779-588-6_10) 22323224 PMC4321859

[68] Xu J, Zhang Y. 2010 How significant is a protein structure similarity with TM-score = 0.5? Bioinformatics **26**, 889–895. (10.1093/bioinformatics/btq066) 20164152 PMC2913670

[69] Davis IW *et al*. 2007 MolProbity: all-atom contacts and structure validation for proteins and nucleic acids. Nucleic Acids Res. **35**, W375–W383. (10.1093/nar/gkm216) 17452350 PMC1933162

[70] Rennie ML, Arkinson C, Chaugule VK, Walden H. 2022 Cryo-EM reveals a mechanism of USP1 inhibition through a cryptic binding site. Sci. Adv. **8**, eabq6353. (10.1126/sciadv.abq6353) 36170365 PMC9519042

[71] Arkinson C, Chaugule VK, Toth R, Walden H. 2018 Specificity for deubiquitination of monoubiquitinated FANCD2 is driven by the N-terminus of USP1. Life Sci. Alliance **1**, e201800162 (doi. (10.26508/lsa.201800162) 30456385 PMC6238601

[72] Rennie ML, Arkinson C, Chaugule VK, Toth R, Walden H. 2021 Structural basis of FANCD2 deubiquitination by USP1−UAF1. Nat. Struct. Mol. Biol. **28**, 356–364. (10.1038/s41594-021-00576-8) 33795880

[73] Ren F *et al*. 2023 AlphaFold accelerates artificial intelligence powered drug discovery: efficient discovery of a novel CDK20 small molecule inhibitor. Chem. Sci. **14**, 1443–1452. (10.1039/d2sc05709c) 36794205 PMC9906638

[74] Yu D, Chojnowski G, Rosenthal M, Kosinski J. 2023 AlphaPulldown—a Python package for protein–protein interaction screens using AlphaFold-Multimer. Bioinformatics **39**, 10–12. (10.1093/bioinformatics/btac749) PMC9805587 36413069

[75] Hilser VJ, Wrabl JO, Motlagh HN. 2012 Structural and energetic basis of allostery. Annu. Rev. Biophys. **41**, 585–609. (10.1146/annurev-biophys-050511-102319) 22577828 PMC3935618

[76] Kryshtafovych A, Montelione GT, Rigden DJ, Mesdaghi S, Karaca E, Moult J. 2023 Breaking the conformational ensemble barrier: ensemble structure modeling challenges in CASP15. Proteins **91**, 1903–1911. (10.1002/prot.26584) 37872703 PMC10840738

[77] del Alamo D, Sala D, Mchaourab HS, Meiler J. 2022 Sampling alternative conformational states of transporters and receptors with AlphaFold2. eLife **11**, e75751. (10.7554/elife.75751) 35238773 PMC9023059

[78] Heo L, Feig M. 2022 Multi-state modeling of G-protein coupled receptors at experimental accuracy. Proteins **90**, 1873–1885. (10.1002/prot.26382) 35510704 PMC9561049

[79] Lazou M, Khan O, Nguyen T, Padhorny D, Kozakov D, Joseph-McCarthy D, Vajda S. 2024 Predicting multiple conformations of ligand binding sites in proteins suggests that AlphaFold2 may remember too much. Proc. Natl Acad. Sci. USA **121**, e2412719121. (10.1073/pnas.2412719121) 39565312 PMC11621821

[80] Stein RA, Mchaourab HS. 2022 SPEACH_AF: sampling protein ensembles and conformational heterogeneity with Alphafold2. PLoS Comput. Biol. **18**, e1010483. (10.1371/journal.pcbi.1010483) 35994486 PMC9436118

[81] Schafer JW, Porter LL. 2023 Evolutionary selection of proteins with two folds. Nat. Commun. **14**, 5478. (10.1038/s41467-023-41237-2) 37673981 PMC10482954

[82] Wayment-Steele HK, Ojoawo A, Otten R, Apitz JM, Pitsawong W, Hömberger M, Ovchinnikov S, Colwell L, Kern D. 2024 Predicting multiple conformations via sequence clustering and AlphaFold2. Nature **625**, 832–839. (10.1038/s41586-023-06832-9) 37956700 PMC10808063

[83] Chakravarty D, Porter LL. 2022 AlphaFold2 fails to predict protein fold switching. Protein Sci. **31**, e4353. (10.1002/pro.4353) 35634782 PMC9134877

[84] Bryant P, Noé F. 2024 Structure prediction of alternative protein conformations. Nat. Commun. **15**, 7328. (10.1038/s41467-024-51507-2) 39187507 PMC11347660

[85] Wallner B. 2023 AFsample: improving multimer prediction with AlphaFold using massive sampling. Bioinformatics **39**, d573. (10.1093/bioinformatics/btad573) PMC10534052 37713472

[86] Schafer JW, Chakravarty D, Chen EA, Porter LL. 2024 Sequence clustering confounds AlphaFold2. bioRxiv. 2024.01.05.574434. (10.1101/2024.01.05.574434) 39972235

[87] Wayment-Steele HK, Ovchinnikov S, Colwell L, Kern D. 2024 A resource for comparing AF-Cluster and other AlphaFold2 sampling methods. bioRxiv. (10.1101/2024.07.29.605333)

[88] Chakravarty D, Schafer JW, Chen EA, Thole JF, Ronish LA, Lee M, Porter LL. 2024 AlphaFold predictions of fold-switched conformations are driven by structure memorization. Nat. Commun. **15**, 7296. (10.1038/s41467-024-51801-z) 39181864 PMC11344769

[89] Schwarz D, Georges G, Kelm S, Shi J, Vangone A, Deane CM. 2021 Co-evolutionary distance predictions contain flexibility information. Bioinformatics **38**, 65–72. (10.1093/bioinformatics/btab562) 34383892

[90] Zhang F, Li Z, Zhao K, Zhao P, Zhang G. 2024 Prediction of inter-residue multiple distances and exploration of protein multiple conformations by deep learning. IEEE/ACM Trans. Comput. Biol. Bioinform. **21**, 1731–1739. (10.1109/TCBB.2024.3411825) 38857126

[91] Anishchenko I, Ovchinnikov S, Kamisetty H, Baker D. 2017 Origins of coevolution between residues distant in protein 3D structures. Proc. Natl Acad. Sci. USA **114**, 9122–9127. (10.1073/pnas.1702664114) 28784799 PMC5576787

[92] Hou M, Jin S, Cui X, Peng C, Zhao K, Song L, Zhang G. 2024 Protein multiple conformation prediction using multi-objective evolution algorithm. Interdiscip. Sci. **16**, 519–531. (10.1007/s12539-023-00597-5) 38190097

[93] Pak MA, Markhieva KA, Novikova MS, Petrov DS, Vorobyev IS, Maksimova ES, Kondrashov FA, Ivankov DN. 2023 Using AlphaFold to predict the impact of single mutations on protein stability and function. PLoS One **18**, e0282689. (10.1371/journal.pone.0282689) 36928239 PMC10019719

[94] Cheng J *et al*. 2023 Accurate proteome-wide missense variant effect prediction with AlphaMissense. Science **381**, eadg7492. (10.1126/science.adg7492) 37733863

[95] Burchell L, Chaugule VK, Walden H. 2012 Small, N-terminal tags activate parkin E3 ubiquitin ligase activity by disrupting its autoinhibited conformation. PLoS One **7**, e34748. (10.1371/journal.pone.0034748) 22496854 PMC3319606

[96] Fatti E, Khawaja S, Weis K. 2025 The dark side of fluorescent protein tagging-the impact of protein tags on biomolecular condensation. Mol. Biol. Cell **36**, br10. (10.1091/mbc.E24-11-0521) 39878648 PMC11974960

[97] Humphreys IR *et al*. 2021 Computed structures of core eukaryotic protein complexes. Science **374**, eabm4805. (10.1126/science.abm4805) 34762488 PMC7612107

[98] Burke DF *et al*. 2023 Towards a structurally resolved human protein interaction network. Nat. Struct. Mol. Biol. **30**, 216–225. (10.1038/s41594-022-00910-8) 36690744 PMC9935395

[99] Bartolec TK, Vázquez-Campos X, Norman A, Luong C, Johnson M, Payne RJ, Wilkins MR, Mackay JP, Low JKK. 2023 Cross-linking mass spectrometry discovers, evaluates, and corroborates structures and protein–protein interactions in the human cell. Proc. Natl Acad. Sci. USA **120**, e2219418120. (10.1073/pnas.2219418120) 37071682 PMC10151615

[100] Sifri C, Hoeg L, Durocher D, Setiaputra D. 2023 An AlphaFold2 map of the 53BP1 pathway identifies a direct SHLD3–RIF1 interaction critical for shieldin activity. EMBO Rep. **24**, e56834. (10.15252/embr.202356834) 37306046 PMC10398656

[101] Weeratunga S *et al*. 2024 Interrogation and validation of the interactome of neuronal Munc18-interacting Mint proteins with AlphaFold2. J. Biol. Chem. **300**, 105541. (10.1016/j.jbc.2023.105541) 38072052 PMC10820826

[102] Homma F, Huang J, van der Hoorn RAL. 2023 AlphaFold-Multimer predicts cross-kingdom interactions at the plant–pathogen interface. Nat. Commun. **14**, 6040. (10.1038/s41467-023-41721-9) 37758696 PMC10533508

[103] Teufel F, Refsgaard JC, Kasimova MA, Deibler K, Madsen CT, Stahlhut C, Grønborg M, Winther O, Madsen D. 2023 Deorphanizing peptides using structure prediction. J. Chem. Inf. Model. **63**, 2651–2655. (10.1021/acs.jcim.3c00378) 37092865

[104] . Danneskiold-Samsøe NB et al. 2024 AlphaFold2 enables accurate deorphanization of ligands to single-pass receptors. Cell Syst. 15, 1046–1060. (10.1016/j.cels.2024.10.004) 39541981 PMC12147870

[105] Fu ZQ, Sha HL, Sha B. 2022 AI-based protein interaction screening and identification (AISID). Int. J. Mol. Sci. **23**, 11685. (10.3390/ijms231911685) 36232986 PMC9570074

[106] Lim Y *et al*. 2023 In silico protein interaction screening uncovers DONSON’s role in replication initiation. Science **381**, eadi3448. (10.1126/science.adi3448) 37590370 PMC10801813

[107] Stahl K, Graziadei A, Dau T, Brock O, Rappsilber J. 2023 Protein structure prediction with in-cell photo-crosslinking mass spectrometry and deep learning. Nat. Biotechnol. **41**, 1810–1819. (10.1038/s41587-023-01704-z) 36941363 PMC10713450

[108] Yin R, Feng BY, Varshney A, Pierce BG. 2022 Benchmarking AlphaFold for protein complex modeling reveals accuracy determinants. Protein Sci. **31**, 1–19. e4379. (10.1002/pro.4379) PMC9278006 35900023

[109] Rout MP, Sali A. 2019 Principles for integrative structural biology studies. Cell **177**, 1384–1403. (10.1016/j.cell.2019.05.016) 31150619 PMC6810593

[110] Mosalaganti S *et al*. 2022 AI-based structure prediction empowers integrative structural analysis of human nuclear pores. Science **376**, eabm9506. (10.1126/science.abm9506) 35679397

[111] Fontana P, Dong Y, Pi X, Tong AB, Hecksel CW, Wang L, Fu TM, Bustamante C, Wu H. 2022 Structure of cytoplasmic ring of nuclear pore complex by integrative cryo-EM and AlphaFold. Science **376**, eabm9326. (10.1126/science.abm9326) 35679401 PMC10054137

[112] Callaway E. 2024 AI has dreamt up a blizzard of new proteins. Do any of them actually work? Nature **634**, 532–533. (10.1038/d41586-024-03335-z) 39407023

[113] Watson JL *et al*. 2023 De novo design of protein structure and function with RFdiffusion. Nature **620**, 1089–1100. (10.1038/s41586-023-06415-8) 37433327 PMC10468394

[114] Frank C *et al*. 2024 Scalable protein design using optimization in a relaxed sequence space. Science **386**, 439–445. (10.1126/science.adq1741) 39446959 PMC11734486

[115] Ingraham JB *et al*. 2023 Illuminating protein space with a programmable generative model. Nature **623**, 1070–1078. (10.1038/s41586-023-06728-8) 37968394 PMC10686827

[116] Bennett NR *et al*. 2023 Improving de novo protein binder design with deep learning. Nat. Commun. **14**, 2625. (10.1038/s41467-023-38328-5) 37149653 PMC10163288

[117] Pacesa M *et al*. 2024 BindCraft: one-shot design of functional protein binders. bioRxiv. (10.1101/2024.09.30.615802) PMC12507698 40866699

[118] Zambaldi V. 2024 De novo design of high-affinity protein binders with AlphaProteo. ArXiv 2409.08022. (10.48550/arXiv.2409.08022)

[119] Sappington I *et al*. 2024 Improved protein binder design using beta-pairing targeted RFdiffusion. bioRxiv. (10.1101/2024.10.11.617496) PMC12852815 41519838

[120] Vázquez Torres S *et al*. 2024 De novo design of high-affinity binders of bioactive helical peptides. Nature **626**, 435–442. (10.1038/s41586-023-06953-1) 38109936 PMC10849960

[121] Rettie SA *et al*. 2023 Cyclic peptide structure prediction and design using AlphaFold. bioRxiv. (10.1101/2023.02.25.529956) PMC12095755 40399308

[122] Kosugi T, Ohue M. 2023 Design of cyclic peptides targeting protein–protein interactions using AlphaFold. Int. J. Mol. Sci. **24**, 13257. (10.3390/ijms241713257) 37686057 PMC10487914

[123] Rettie SA *et al*. 2024 Accurate de novo design of high-affinity protein binding macrocycles using deep learning. bioRxiv (10.1101/2024.11.18.622547) PMC12643943 40542165

[124] Dauparas J *et al*. 2022 Robust deep learning–based protein sequence design using ProteinMPNN. Science **378**, 49–56. (10.1126/science.add2187) 36108050 PMC9997061

[125] Goverde CA *et al*. 2024 Computational design of soluble and functional membrane protein analogues. Nature **631**, 449–458. (10.1038/s41586-024-07601-y) 38898281 PMC11236705

[126] Adaptyv Biosystems Inc. 2024 Protein design competition: has binder design been solved?. See https://www.adaptyvbio.com/blog/po104.

[127] Raouraoua N, Lensink MF, Brysbaert G. 2025 Massive sampling strategy for antibody–antigen targets in CAPRI Round 55 with MassiveFold. Proteins(10.1002/prot.26802) PMC13648903 39868877

[128] Kryshtafovych A, Schwede T, Topf M, Fidelis K, Moult J. 2023 Critical assessment of methods of protein structure prediction (CASP)—Round XV. Proteins **91**, 1539–1549. (10.1002/prot.26617) 37920879 PMC10843301

[129] Bernard C, Postic G, Ghannay S, Tahi F. 2024 Has AlphaFold 3 reached its success for RNAs? bioRxiv. (10.1101/2024.06.13.598780)

[130] Zhang C, Zhang Y, Pyle AM. 2023 rMSA: a sequence search and alignment algorithm to improve RNA structure modeling. J. Mol. Biol. **435**, 167904. (10.1016/j.jmb.2022.167904) 37356900

[131] Inukai S, Kock KH, Bulyk ML. 2017 Transcription factor–DNA binding: beyond binding site motifs. Curr. Opin. Genet. Dev. **43**, 110–119. (10.1016/j.gde.2017.02.007) 28359978 PMC5447501

[132] Martinez-Goikoetxea M. 2024 CCfrag: scanning folding potential of coiled-coil fragments with AlphaFold. Bioinform. Adv. **5**, vbae195. (10.1093/bioadv/vbae195) 39735573 PMC11676326

[133] Lee CY *et al*. 2024 Systematic discovery of protein interaction interfaces using AlphaFold and experimental validation. Mol. Syst. Biol. **20**, 75–97. (10.1038/s44320-023-00005-6) 38225382 PMC10883280

[134] Kallenborn F, Chacon A, Hundt C, Sirelkhatim H, Didi K, Dallago C, Mirdita M, Schmidt B, Steinegger M. 2024 GPU-accelerated homology search with MMseqs2. bioRxiv. (10.1101/2024.11.13.623350) PMC12510879 40968302

[135] Barrio-Hernandez I *et al*. 2023 Clustering predicted structures at the scale of the known protein universe. Nature **622**, 637–645. (10.1038/s41586-023-06510-w) 37704730 PMC10584675

